# Psychisch gesunde Kinder: eine gesamtgesellschaftliche Herausforderung

**DOI:** 10.1007/s00103-023-03731-2

**Published:** 2023-07-07

**Authors:** Uwe Koch-Gromus, Joseph Kuhn

**Affiliations:** 1 Institut und Poliklinik für Medizinische Psychologie, Universitätsklinikum Hamburg Eppendorf, Martinistraße 52, 20246 Hamburg, Deutschland; 2 Bayrisches Landesamt für Gesundheit und Lebensmittelsicherheit, Veterinärstraße 2, 85764 Oberschleißheim, Deutschland

Viele Kinder erfreuen sich einer guten Gesundheit, körperlich wie psychisch. Allerdings sind auch Entwicklungsstörungen und psychische Probleme bei Kindern keine Ausnahme. Lange Zeit gab es dazu in Deutschland keine repräsentativen Daten. Das hat sich inzwischen grundlegend geändert. Etwa ein Fünftel der 3‑ bis 17-Jährigen waren der KiGGS (Studie zur Gesundheit von Kindern und Jugendlichen in Deutschland)-Studie zufolge psychisch auffällig, mit leicht rückläufiger Tendenz von der KiGGS-Basiserhebung 2003–2006 zur KiGGS Welle 2 im Zeitraum 2014–2017. Die Coronakrise hat diese Entwicklung umgekehrt und zu einer deutlichen Zunahme psychischer Auffälligkeiten auch bei Kindern geführt, wie die COPSY (COrona und PSYche)-Studie zeigt.

Die Verfügbarkeit von Daten zur psychischen Gesundheit von Kindern, einschließlich der positiven Aspekte psychischen Wohlbefindens, soll in den kommenden Jahren weiter ausgebaut werden. Es ist vorgesehen, die nationale Mental Health Surveillance (MHS) am Robert Koch-Institut, die bisher auf das Erwachsenenalter fokussiert war, auf Kinder und Jugendliche auszuweiten, so dass auch für diese Lebensphase Indikatoren zur Verfügung stehen.

Bei der psychischen Gesundheit von Kindern ist häufig keine strikte Trennung von körperlichen und psychischen Aspekten möglich. Körperliche Beeinträchtigungen haben in diesem Alter häufig psychische Begleiterscheinungen, umgekehrt gehen auch psychische Erkrankungen häufig mit körperlichen Symptomen einher. Ein weiterer wichtiger Punkt ist, dass psychische Störungen bei Kindern praktisch immer auch das soziale Umfeld tangieren, Eltern, Geschwister, Kita oder Schule, und dass dies für die Therapie von essentieller Bedeutung ist und darüber hinaus auch präventive Relevanz hat.

Von den Diagnosen her gesehen, stehen bei kleinen Kindern Entwicklungsstörungen im Vordergrund, im Schulalter dann vermehrt ADHS, Störungen des Sozialverhaltens, Zwänge, depressive Symptomatiken und Ängste, bei älteren Kindern, im Übergang zum Jugendalter, auch schon Essstörungen, Suchtmittelmissbrauch und Suizidalität.

Die Daten ziehen Taten nach sich: Die ambulante Versorgung ist in den letzten Jahren deutlich ausgebaut worden, es gibt viele schulische und kommunale Präventionsprojekte für Kinder, auch erfolgreich verstetigte Strukturen, wie z. B. die „Frühen Hilfen“ (https://www.fruehehilfen.de). Zugleich besteht aber auch weiterhin großer Handlungsbedarf. Einige Aspekte werden in diesem Heft aufgegriffen, verbunden mit dem Ziel, die Wahrnehmung der Wichtigkeit dieses Themas weiter zu befördern. Ein späteres Heft soll dann auch die psychische Gesundheit von Jugendlichen behandeln.

Die ersten 3 Beiträge behandeln Grundlagen der psychischen Gesundheit und kindlichen Entwicklung. *S. Walper et al.* setzen sich mit belastenden Kindheitserfahrungen (Adverse Childhood Experiences) und deren Auswirkungen auf die Emotionalität von Kindern im Säuglings- und Kleinkindalter auseinander. Ihre Ergebnisse entsprechen internationalen Befunden und bestätigen die Bedeutung eines gut ausgebauten Systems „Früher Hilfen“. *F. Reiß et al.* berichten auf der Grundlage von 3 epidemiologischen Studien Ergebnisse zum seelischen Wohlbefinden von Kindern und Jugendlichen vor und während der COVID-19-Pandemie. Gegenüber der präpandemischen Situation verschlechterte sich nach Beginn der COVID-19-Pandemie zunächst die psychische Gesundheit, 2 Jahre später zeigen sich zwar Verbesserungen, aber das Ausgangsniveau vor der Pandemie wird – außer bei depressiven Symptomen – noch nicht wieder erreicht. Es besteht ein besonderer Bedarf an Unterstützungsmaßnahmen infolge der Pandemie. In einem Übersichtsartikel stellen *U. Thyen et al.* die biopsychosozialen Zusammenhänge bei kindlichen Gesundheitsstörungen und Beeinträchtigungen dar, wobei sie v a. die Entwicklungsneurologie in den Blick nehmen.

Die folgenden 5 Beiträge befassen sich mit der Behandlung und Versorgung von psychischen Problemen im Kindesalter. *R. Schepker und M. Kölch* analysieren die strukturellen Bedingungen der kinder- und jugendpsychiatrischen Versorgung. Sie verweisen insbesondere auf den deutlichen Zuwachs bei niedergelassenen Praxen, Institutsambulanzen und tagesklinischen Behandlungsplätzen und kritisieren Defizite beim Angebot stationsäquivalenter Behandlung (StäB) und in der sektorenübergreifenden Versorgung. *M. Ziegler et al. *beschreiben eindrucksvoll die hohe psychische Belastung von Familien bei frühkindlichen psychischen Störungen, insbesondere bei Kindern mit Schlaf- und Fütterstörungen. Dargestellt wird ein wissenschaftlich begründetes niedrigschwelliges Angebot für die Familien, um Vernachlässigungen, Misshandlungen und psychischen Folgeerkrankungen des Kindes vorzubeugen. *M. Gerlach et al. *beschreiben die besonderen Voraussetzungen einer psychopharmakologischen Behandlung bei Kindern und Jugendlichen und gehen dabei auf Fragen zur altersgerechten Einbeziehung der Kinder in den Entscheidungs- und Aufklärungsprozess, auf Schwierigkeiten des Wirknachweises sowie auf haftungsrechtliche Fragen bei der Off-Label-Anwendung ein. *G. Schulte-Körne et al. *analysieren den Revisionsprozess bei der 2013 erstmals veröffentlichten deutschsprachigen Leitlinie zur Behandlung depressiver Störungen bei Kindern und Jugendlichen. Eine veränderte Evidenzlage ergibt sich vor allem in Bezug auf einige Antidepressiva und sportliche Aktivitäten.

Im abschließenden Themenbereich Förderung psychischer Gesundheit durch Präventions- und Gesundheitsförderungsmaßnahmen finden sich 4 Beiträge, die recht unterschiedliche Aspekte und Konzepte aufgreifen. Zunächst stellen* U. Walter et al. *im Kontext des internationalen Präventionssystems „Communities That Care“ (CTC) einen kommunalen Ansatz der Gesundheitsförderung dar. Das Konzept wurde in den USA entwickelt und auf deutsche Rahmenbedingungen übertragen. Die vernetzte Mehrebenenstrategie zielt im kommunalen Kontext auf die Prävention von Alkohol- und Drogenmissbrauch, Gewalt, Delinquenz, Schulabbruch und depressiven Symptomen bei Heranwachsenden. CTC integriert als systemische Intervention vorhandene örtliche Strukturen und Organisationen und bindet diese über neue Entscheidungs- und Entwicklungsgremien in den gesamten Prozess ein. *H. L. Jörren et al. *berichten Ergebnisse einer Sekundäranalyse zu Einflussfaktoren der psychischen Gesundheit im Kindesalter und fokussieren dabei die Aspekte Mediennutzung, Erziehungsverhalten und elterliches Stresserleben. Die Datenanalyse auf der Basis mehrerer nationaler epidemiologischer Untersuchungen im Kindesalter unterstreicht die besondere Bedeutung des elterlichen Verhaltens und relativiert zumindest partiell das besondere Risiko einer Gefährdung der psychischen Gesundheit durch hohe Mediennutzung. *R. Schütz und L. Bilz *untersuchen die Bedeutung von Einsamkeit bei 11- bis 15-jährigen deutschen SchülerInnen. Circa jede/r fünfte SchülerIn beschreibt sich als „meistens“ oder „immer“ einsam, häufiger trifft dies auf Mädchen und auch auf Kinder mit niedrigerem sozialen Status zu. Insgesamt sind allerdings die empirischen Grundlagen zu diesem wichtigen Thema noch defizitär. *U. Gebhard *gibt eine Übersicht über Effekte von Naturerfahrung bei Kindern. Der Autor interpretiert die Befunde als Beleg dafür, dass Naturerfahrungen gesundheitsfördernde Wirkungen bei Kindern haben.

Wir hoffen, mit diesem Themenheft zur psychischen Gesundheit im Kindesalter eine gute und inhaltlich anregende Auswahl von Beiträgen getroffen zu haben. Wir danken allen AutorInnen für die sehr gute Zusammenarbeit bei der Erstellung des Themenheftes und wünschen Ihnen, liebe LeserInnen, eine anregende Lektüre.

